# Supplementary figures and images for: The growth factor BMP11 is required for the development and evolution of a male exaggerated weapon and its associated fighting behavior in a water strider
Source: PLoS Biol. 2021 May 11;19(5):e3001157. doi: 10.1371/journal.pbio.3001157 (PMC8112723; doi:10.1371/journal.pbio.3001157)

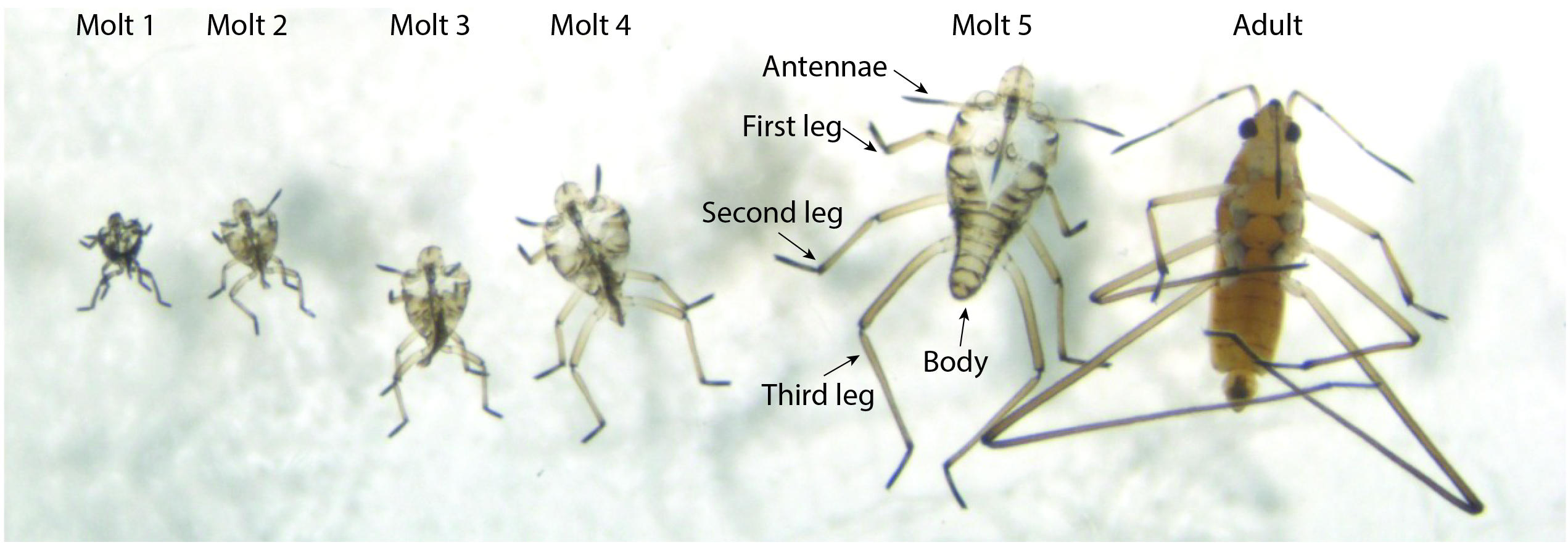

Supplement: S1 Fig — These molts were used to build a growth curve for each individual during nymphal development. (TIF) [file pbio.3001157.s001.tif]

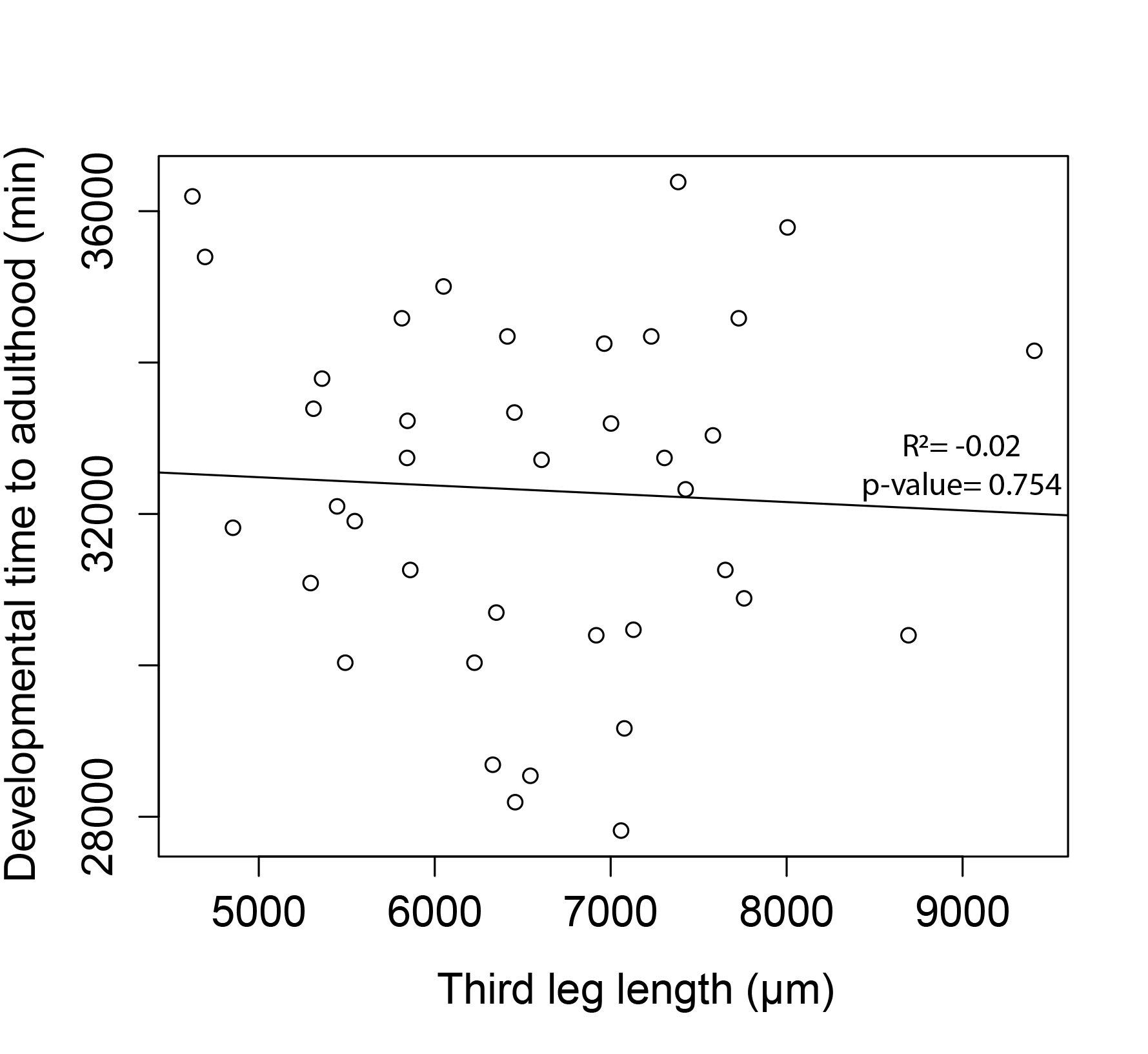

Supplement: S2 Fig — R-squared and p-value of the linear regression are indicated. The data underlying this figure may be found at https://datadryad.org/stash/share/2Q9tb7wD4R_iWkDSRAiOY2NsFuXCZE99o60C6Oeydk4. (TIF) [file pbio.3001157.s002.tif]

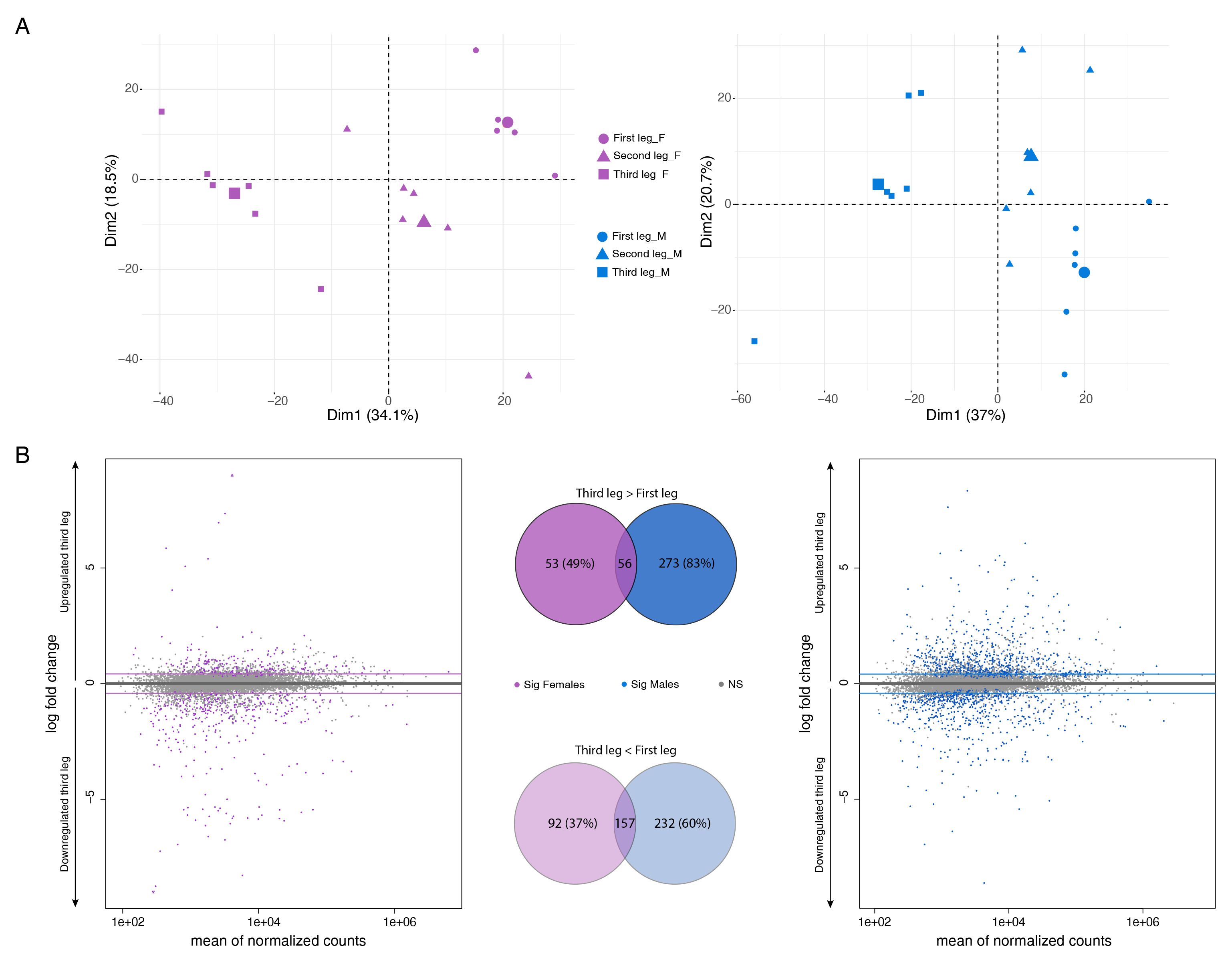

Supplement: S3 Fig — (A) PCA analysis of expressed genes in male and female legs separately. (B) MA plots of transcripts differentially expressed in the third and first legs in both sexes. Gray circles represent unbiased genes, while colored circles (blue in males, purple in females) represent genes significantly differentially expressed between the 2 pairs of legs at an adjusted p-value of <0.05. Venn diagrams illustrate the number of leg-biased genes (fold-change > 1.5) shared in females (purple) and males (blue), both for up-regulated genes in the third (top) and first legs (down). The data underlying this figure may be found at https://datadryad.org/stash/share/2Q9tb7wD4R_iWkDSRAiOY2NsFuXCZE99o60C6Oeydk4. MA, Log ratio and Mean average; NS, Non significant; PCA, Principal component analysis. (TIF) [file pbio.3001157.s003.tif]

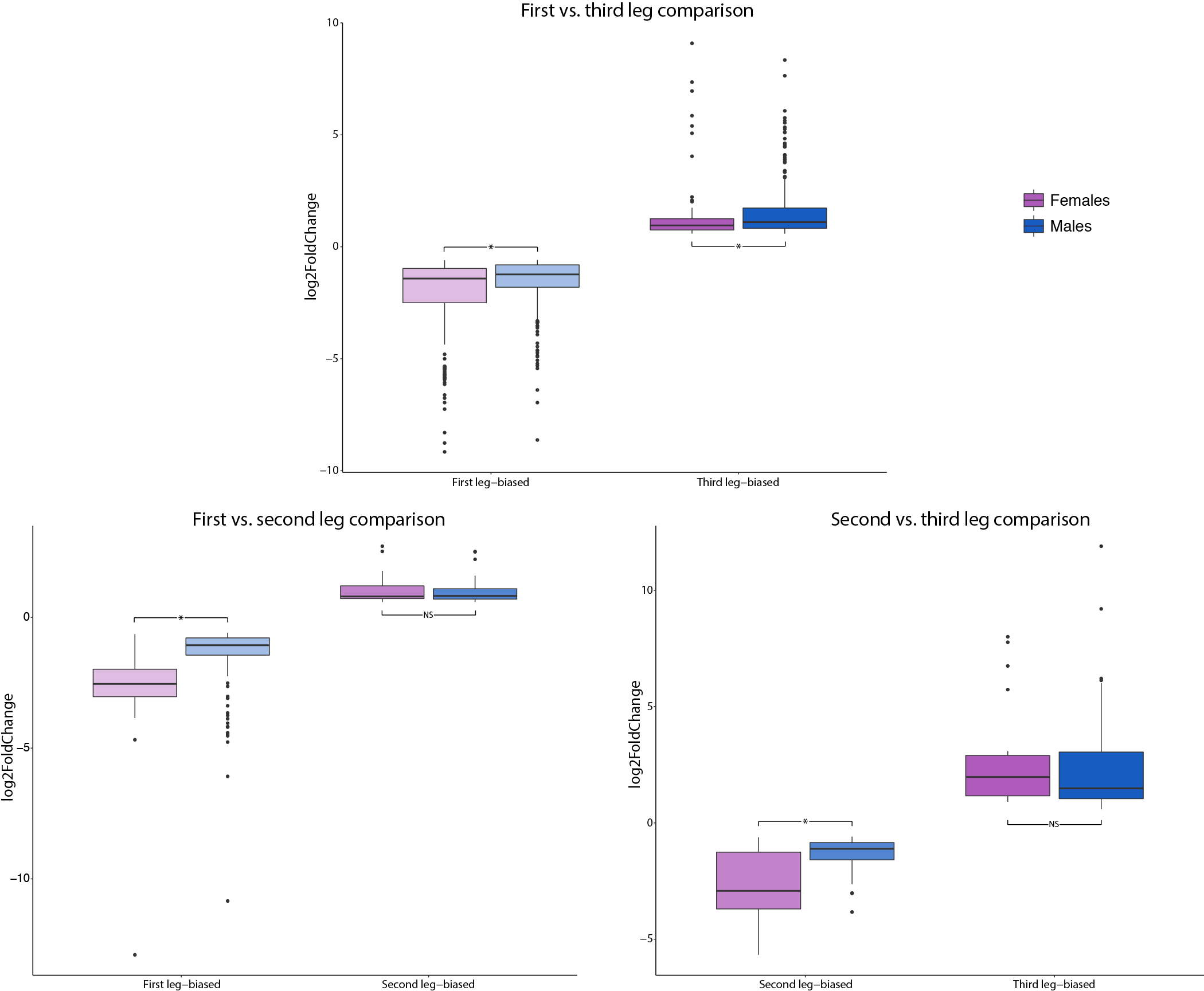

Supplement: S4 Fig — The data underlying this figure may be found at https://datadryad.org/stash/share/2Q9tb7wD4R_iWkDSRAiOY2NsFuXCZE99o60C6Oeydk4. (TIF) [file pbio.3001157.s004.tif]

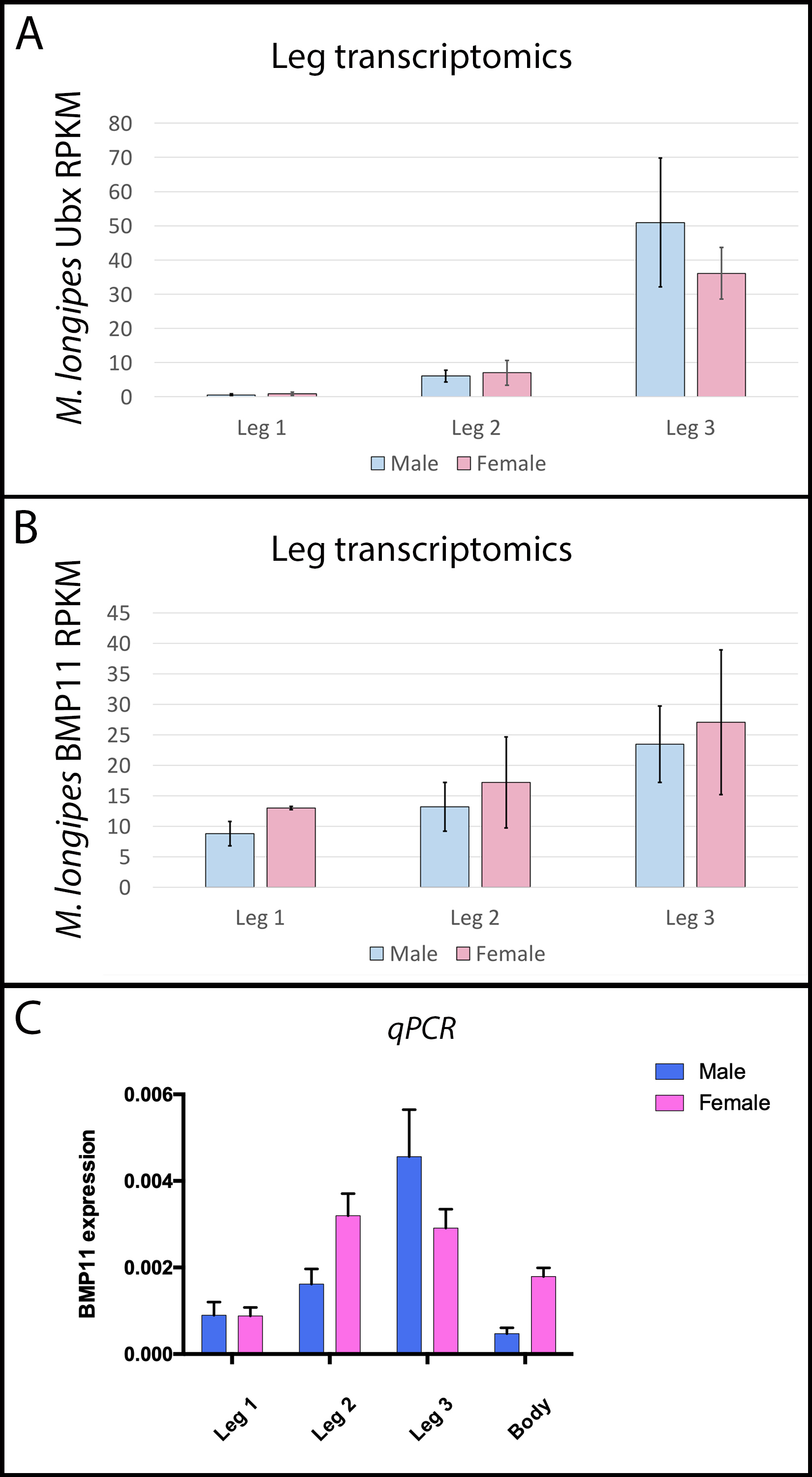

Supplement: S5 Fig — Levels of expression of Ubx and BMP11 transcripts in M. longipes as revealed by comparative transcriptomics (A and B) and by quantitative RT-PCR (C). Note that for qPCR we also determined that BMP11 is significantly biased in female body (two-way ANOVA; p < 0.0001). The data underlying this figure may be found in S10 and S11 Tables. BMP11, Bone Morphogenetic Protein 11; RT-PCR, reverse transcription polymerase chain reaction; Ubx, Ultrabithorax. (TIF) [file pbio.3001157.s005.tif]

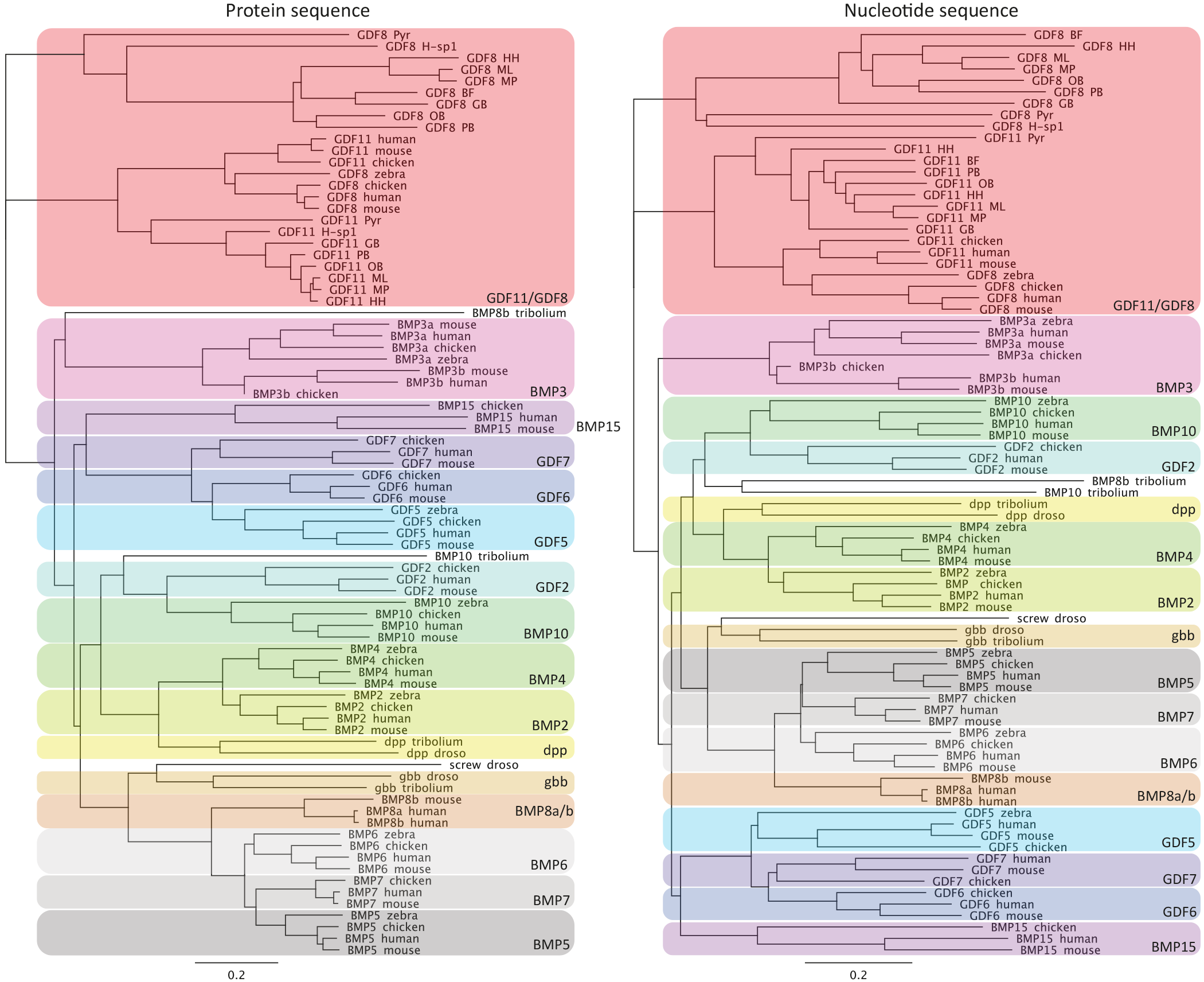

Supplement: S6 Fig — BF: Brachymetra furra, HH: Husseyella halophyla, ML: Microvelia longipes, MP: Microvelia pulchella, OB: Oiovelia brasiliensis, PB: Platyvelia brachialis, GB: Gerris buenoi, Pyr: Pyrrhocoris apterus, Tribolium: Tribolium casteneum, Droso: Drosophila melanogaster, H.sp1: Hebrus sp1, chicken: Gallus gallus, human: Homo sapiens, mouse: Mus musculus. Nucleotide and protein alignment files can be found in S1 and S2 Data. The data underlying this figure may be found at https://datadryad.org/stash/share/2Q9tb7wD4R_iWkDSRAiOY2NsFuXCZE99o60C6Oeydk4. (TIF) [file pbio.3001157.s006.tif]

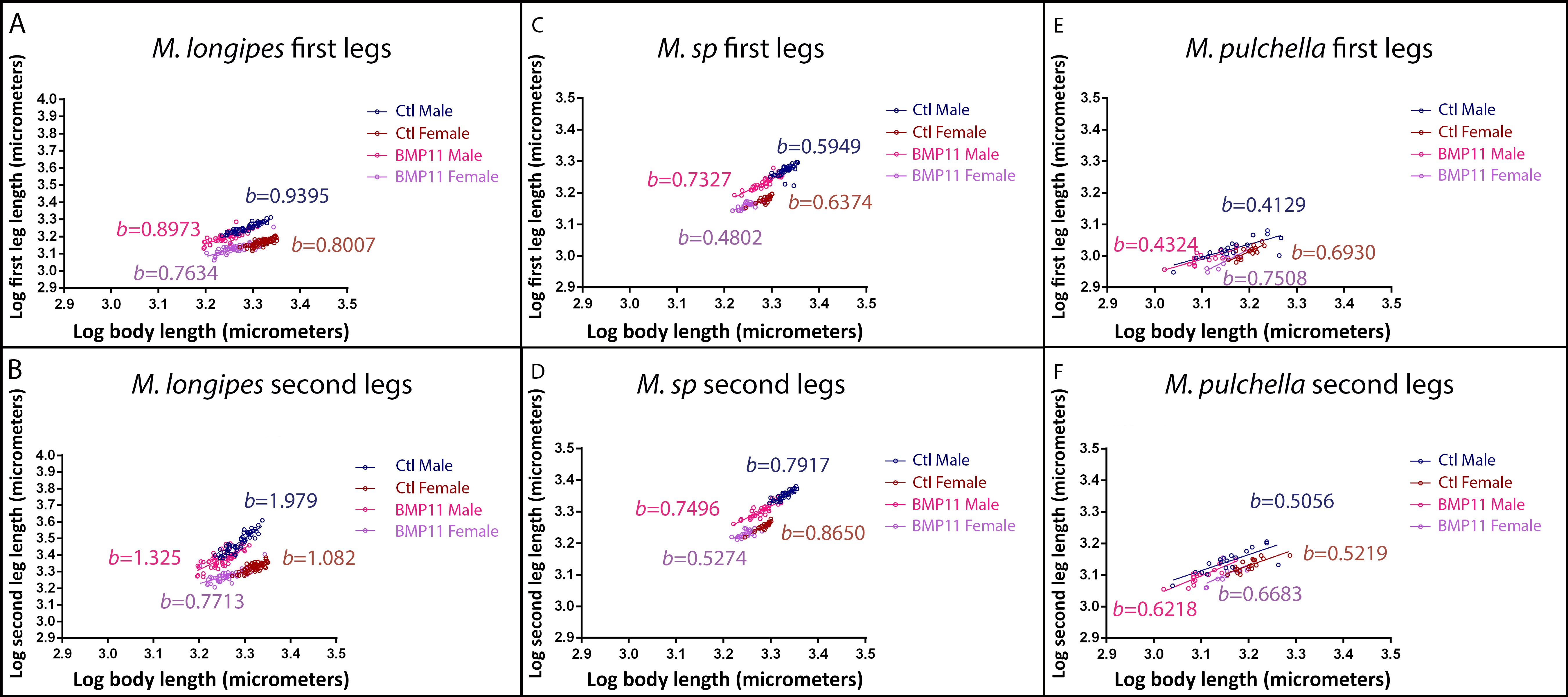

Supplement: S7 Fig — There is no effect of BMP11 (GDF11) RNAi on slope in any of these tissues except the exaggerated second legs of M. longipes (B). The data underlying this figure may be found in S6 and S7 Tables. BMP11, Bone Morphogenetic Protein 11; Ctl, Control; GDF11, Growth Differentiation Factor 11; RNAi, RNA interference. (TIF) [file pbio.3001157.s007.tif]

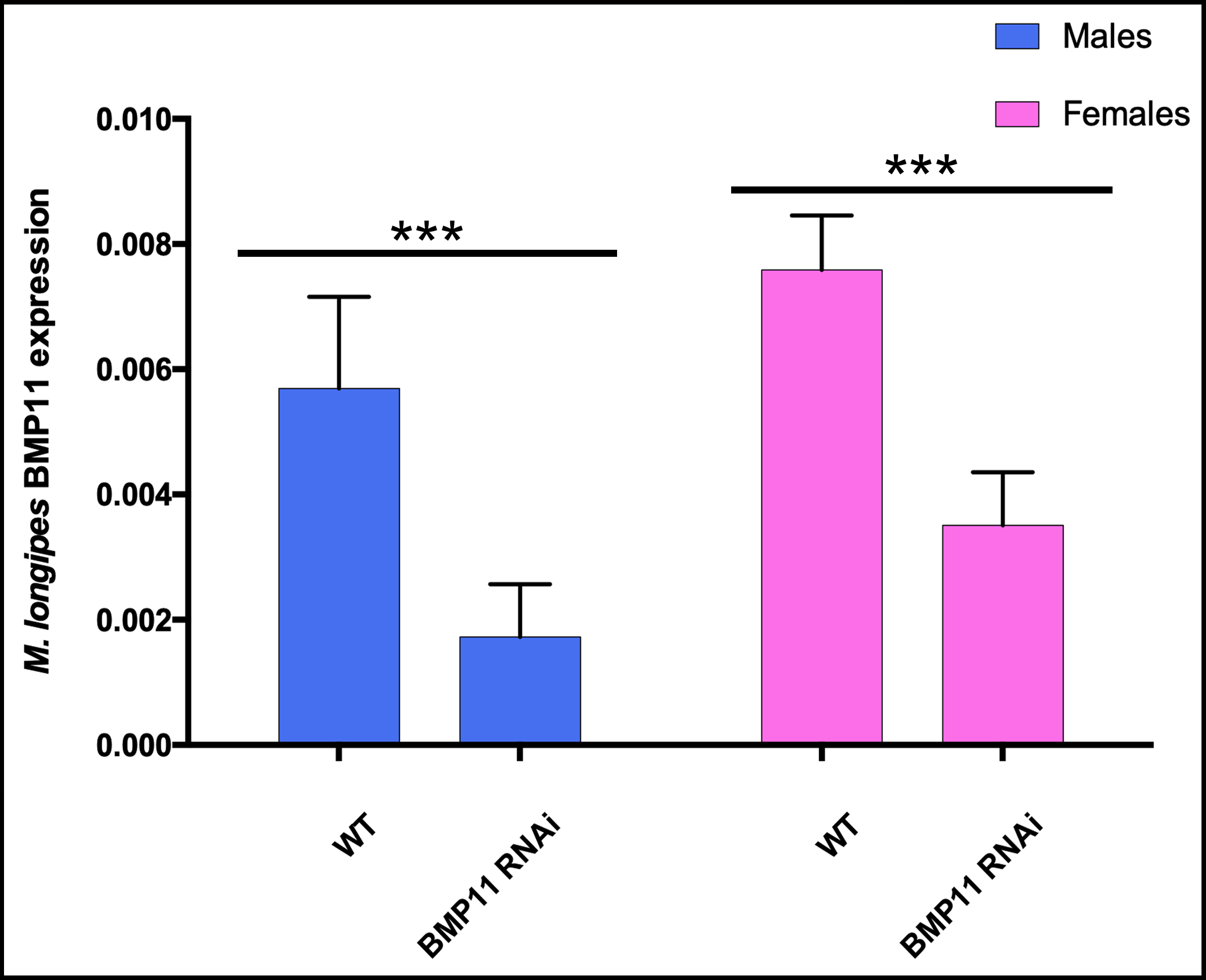

Supplement: S8 Fig — The data underlying this figure may be found in S12 Table. BMP11, Bone Morphogenetic Protein 11; RNAi, RNA interference; RT-PCR, reverse transcription polymerase chain reaction; WT, wild type. (TIF) [file pbio.3001157.s008.tif]
