# Supplementary material for: The growth factor BMP11 is required for the development and evolution of a male exaggerated weapon and its associated fighting behavior in a water strider
Source: PLoS Biol. 2021 May 11;19(5):e3001157. doi: 10.1371/journal.pbio.3001157 (PMC8112723; doi:10.1371/journal.pbio.3001157)
Supplement: Attachment — Submitted filename: Response_to_Reviewers_Jan15_2021.docx [file pbio.3001157.s026.docx]

**Reviewer #1**

(Arnaud Le Rouzic, signed): Review of "Pleiotropy promotes male exaggerated weapon and its associated fighting behaviour in a water strider" by Dr Toubiana and colleagues, PBIOLOGY-D-20-00810R1.

This manuscript reports an interesting set of results on the genetic architecture of the leg length in a species of water strider. In this species, the third pair of legs is much longer in males than in other related species, which is strongly suggestive of sexual selection. The authors show that (i) the leg length difference is due to a change in the limb development at the end of the nymphal stage, (ii) a set of genes which expression is associated with the third legs length could be identified, (iii) two genes, BMP11 and Ubx, could be validated by RNAi knockdown; the knockdown had a strong effect in allometric slope for BMP11 but not for Ubx; (iv) RNAi genotypes were characterised by a different fighting behaviour. The authors conclude that this interaction pattern between body size, leg length, and pleiotropy could explain the emergence of this specific, sexually selected trait.

I am rather balanced about the manuscript. In the one hand, I found the result on the genetic architecture of allometric slopes very interesting. Whether or not allometry participates to the selection response (and even whether or not it is evolvable at all) remains an open question, and the results presented in the paper suggest that allometry is indeed evolvable, and that in addition allometric slopes and intercepts might be affected by different genes. As far as I can tell, this genuinely improves our understanding of the genetic bases of allometric relationships. The evidence based on RNAi "only" (not underestimating the complexity of genetic studies in non-model organisms) might need to be confirmed independently, but this seems to match perfectly the scope of Plos Biology short reports ("Short Reports are research articles that may be preliminary, based on a small number of experiments that might not completely flesh out the biological phenomenon under study"). On the

other hand, the manuscript suffers from numerous weaknesses and inaccuracies, some being benign, others being more embarrassing, up to make peer-review uncomfortable due to the lack of information (or to the lack of consistency between different sources of information).

*** Major concerns ***

1. I have the feeling that the manuscript may have been deeply re-organised several times. This is not a problem per se, but the reader often lacks basic information to understand the authors' reasoning. Moreover, the manuscript abuses references to a companion paper, which was provided as supplementary material.

Yet, this reference should not preclude self-consistency of the current manuscript, and I was rather confused about the lack of basic information about the experimental protocol (how many individuals measured? What is the biological material (population, inbred lines, wild-caught vs lab-raised individuals? There are a couple of references to selected lines from ref 19, line 270 mentions "the two inbred populations" (?), this was genuinely confusing).

How many populations / samples / replicates for the transcriptome analysis? Line 295 refers to "controls" (what are they?).

***Authors’ response:***

- *Two papers were initially submitted back to back to PLoS Biology (a genome paper and this one). Unfortunately only the current one was sent for review. As the two papers were related and overlapped in the comparative transcriptomics section, we wanted the editor and reviewers to see both out of transparency and judge for themselves whether they would be ok with the overlap. This is no longer the case now and we have made sure the current revision stands alone from the other paper. So, the problem of referencing between the two papers no longer exists.*
- *We added the missing details in the “Material and methods” section so the reader obtains all information needed. We now specify that the individuals used for the growth curve analyses belong to a lab stock population collected from fieldwork. We give details about the experimental design used in the comparative transcriptomic approach (i.e., type of populations used, the tissues used for RNA extraction, the type of comparisons taken into account in our analyses, the number of individuals per replicate and the number of replicates per comparison). We also replaced most of the transcriptomics data by new experimental data to support our conclusion about the evolution of slope and to keep the story focused. This also helped remove redundancy with the other paper.*
- *For controls, these are either individuals, from the same cohort as RNAi individuals, that were injected with buffer or non-injected. The experiment is set such that control and RNAi individuals are reared in parallel and fed ad libitum daily. These controls along with additional explanations of how we confirmed RNAi effect and reproducibility are now detailed in the methods section with reference to new figures in the supplement.*

Even more embarrassingly, I could not find any of the supplementary tables (in the supplementary PDF, there are 11 sup figures, and the captions of the supplementary tables and movies; I had a link to the movies but not to the supplementary tables), and the figure resolution was so low in the main document that I could barely read the captions.

When digging into the companion paper or counting the data points in the figures, sample sizes and replication conditions seem adequate in most cases, so I don't think that the authors are hiding details on purpose; I assume that this rather comes from copy-paste etc. across several papers without checking that the necessary information was provided.

***Authors’ response:***

- *When the genome (companion) paper was rejected from PLoS Biology based on editorial decision, we submitted it elsewhere and the supplementary tables mistakenly went with it. This was an unfortunate incident. PLoS Biology notified us that the tables were missing on April 29, and we sent them the same day, but it seems they still couldn’t reach the reviewers. They are now available with the revision.*
- *For the resolution of the figures, we submit the highest resolution tiff files that the submission system can accept. Those are formatted automatically by the submission system to generate the pdf file, but this file gives the reviewers a link to download the high-resolution original files if they wish (top right corner of figure page). We encourage the reviewer to take advantage of this option for the current revision.*

Paradoxically, the manuscript gave a fair amount of unnecessary details (such as the focal of the camera for the behavior movies) without making the experiment reproducible (how large were the artificial puddles, what distance was considered to define that the fight occurred "near a floater", how long were the recordings?).

***Authors’ response:*** *We now added more details as per the reviewer’s recommendation. We also removed the term “near a floater” which, we agree is vague. What the reviewer sees in video1 is highly typical. A male stands on the floater and signals through ripples to attract females. A challenging male approaches, and the fight starts where the two males kick each other with the third legs. We specified information about video recording and added two Supplementary tables that provide the record of the number and duration of all fights (Supplementary tables 8 and 9). We also give a summary description of the video record timing, average fight duration and fighting frequency for each replicate (Supplementary tables 8 and 9).*

Note that the statistics are not reproducible

(I did not find any link to the code, and key analyses (such as the power-law fit) was not documented beyond the fact that the authors used RStudio).

***Authors’ response:*** *We have now uploaded everything needed to reproduce the analyses to dryad. Those can be accessed using the following link:*

[https://datadryad.org/stash/share/2Q9tb7wD4R_iWkDSRAiOY2NsFuXCZE99o60C6Oeydk4](https://datadryad.org/stash/share/2Q9tb7wD4R_iWkDSRAiOY2NsFuXCZE99o60C6Oeydk4)*.*

2. Allometry is the central topic of the paper (and I think the title should be changed, see below). Yet, the introduction does not introduce basic facts about allometry; the reader is thus expected to be familiar with the manipulation of allometric slopes and intercepts, which may not match the requirements of a wide-audience journal.

***Authors’ response:*** *We added a paragraph in the introduction that addresses this concern.*

In addition, some sentences appear to be very confusing, e.g. line 51, "a genetic link between [...] traits and other sexual traits such as male body size": isn't it exactly what allometry is about? , or line 62, "the evolution of hyperallometry may be linked to the evolution in increased mean [...] body size": does this make sense?.

***Authors’ response:***

- *We have now clarified these statements.*
- *Regarding the question of allometry among Microvelia spp., we published a paper in 2019 (Toubiana and Khila Proc. B) showing that leg size and body size could co-evolve or evolve independently in the genus Microvelia, which is manifested in different evolutionary patterns of allometry. Furthermore, we have shown that body size responded dramatically to diet but slope (leg 3 by body) did not (Toubiana and Khila 2019). There are species (e.g. M. pulchella) with high variation but small mean body size that did not evolve hyperallometry. Others (e.g. M. sp) have large mean body size and little variation, but also did not evolve hyperallometry. The current study shows that some genes, like Ubx, can change leg length without affecting body size. A recent study by others (Yasukazu Okada et al. PLoS Biol. 2019) demonstrated that some Insulin-like peptides can change the allometric coefficient of a secondary sexual trait in a beetle without changing body size. Finally, our study shows for the first time that some genes, like BMP11, likely have pleiotropic functions and regulate allometric coefficient, body size and male aggressiveness. Our new data (Figure 3) show that while the role of BMP11 in increasing body size is ancestral and non sexually dimorphic in the Microvelia species we tested, its role in increasing slope is specific to M. longipes. This shows that the legs in M. longipes newly acquired this genetic correlation with the body.*

To be honest, I thought at that point that the authors were simply confused about what allometry is about; Details in the methods and in some figures made me changed my mind; at least, allometry was probably measured properly, and discussed in a reasonable way in some parts of the manuscript. Yet, the authors made the strange choice of working on the trait scale and fit non-linear (power law) model, which makes everything more complicated and more difficult to interpret. Why not running the whole analysis on log traits? This would (partly) fix the heteroscedasticity that is obvious from figure 3A and 3C, make allometric relatioships linear, make it possible to use a traditional linear models ( ~ sex + leg + genotype + interactions) to compare allometric slopes and/or intercepts with t-tests...

***Authors’ response:*** *Figures were made with trait scale and fit with power law regressions to show what the actual effects of the knockdowns were on the individuals. However, as explained in the material and methods, all statistical analyses to compare differences in allometric parameters such as intercepts or slopes, were performed on log transformed data. To avoid any misunderstanding, we now show log transformed data in the new figures (including supplementary figures). Instead of using t-tests we ran an ANCOVA with body size as covariate which are statistics specifically used to compare allometric parameters and which are commonplace for this type of analyses, see (Emlen et al. Science 2012; Yasukazu Okada et al. PLoS Biol. 2019).*

If necessary, the figure axes could be log axes so that the original meaning of the trait measures is not lost. At least, working on a log scale would have avoided what is probably an interpretation error of fig 1B, as the authors observe "a dramatic increase in leg length [...] between the 5th instar and adult males" (line 81) (from the figure: from about 3000 to 6000 µm). However, the length at the 4th instar seems to be about 1500 µm, which would mean that the leg length is simply doubling

betweeen larval stages, including the last transition, and that there is nothing specific about this particular developmental stage.

***Authors’ response:***

*The “dramatic increase in leg length” mentioned in the text refers to several comparisons:*

1. *Between 5^th^ instars and adult stages for the third legs.*
2. *When we compare the increase in leg growth between males and females, between the 5^th^ and adult stages.*
3. *When we compare the increase in leg growth between male first and third legs, between the 5^th^ and adult stages.*

*All these comparisons look at only one quantitative trait (leg length) over nymphal stages which therefore does not require log-transformations. We did log-transforme the data, though, when we analyzed ontogenetic allometries since we looked at the relationships between two quantitative traits (e.g. L3 vs L1) over nymphal development.*

*To address the reviewer’s comment, we reanalyzed the data by looking only at mean values of leg length within each nymphal stage and calculating the ratio of these mean values between two successive nymphal stages. Summary results are shown in “Supplementary tables 1”. We can see that male third legs increase 1.78 times in length between the 4rth and the fifth nymphal instar, and 2.18 times in length between the 5th instar and the adult stage. This, in addition to the ease of working with the 5th instar, confirms that we made the right choice by focusing on this instar. Moreover, we could compare these ratios with female third legs and male first legs. In these two comparisons we show that the ratio between the 5^th^ instar and adult stages is first, much smaller than the one in male third legs (1.43 and 1.42 for the female and male legs, respectively). Second, this ratio is not much different from the other ratios comparing earlier developmental stages (1.28 and 1.26 for the female and male legs between the first and second instars, respectively). In contrast, ratios in male third legs go from 1.28 between the first and second instars to 2.18 between the 5^th^ instar and adult stage. Altogether, these results confirm the dramatic increase in leg length of male third legs at the last developmental stage.*

*We modified the sentence to make our message clearer to the reader: “A dramatic increase in leg length was observed between 5th instar and adult males (Figure 1B).” was modified into “A dramatic increase in the third leg length was observed between 5th instar and adult males, but also when compared to females or other male legs during this last developmental stage (Figure 1B).”*

3. The manuscript reports three experimental results, which have different strengths.

(i) The RNAi + length measurement experiment seems the most convincing, even if it probably lacks replication. (ii) The behavioral experiment is also convincing, although here again the possibility that the causal effect of RNAi is not really demonstrated. (iii) The transcriptomics analysis is very descriptive (and probably very noisy), and I am not sure it brings a lot of understanding to the main question. It might be that the only interesting result of the transcriptomics approach to the question raised in the introduction is to shortlist a set of genes that are overexpressed specifically in male third legs, but is remains unclear whether the list of genes from sup table 5 was derived from the transcriptomics approach. To be fair, reporting transcriptomics results that does not seem to be informative nor reliable is the norm in the field, but I found the ratio explanatory power/effort to understand fig 2 particularly disappointing.

***Authors’ response:***

- *These experiments are interconnected. We couldn’t identify genes involved in allometry without the transcriptome, and we couldn’t test behavior without identifying and knocking down genes. Nevertheless, we agree that the transcriptomics section breaks the flow of the story. At the request of other reviewers too, we now removed the detailed analyses to focus on the big picture, which is the characterization and description of the leg-biased genes that will be further used for generating the shortlist of candidate genes and performing the functional assays.*
- *For RNAi, we have conducted multiple experiments to make sure that it is efficient (qPCR), reproducible (injecting dsRNA from three pieces of BMP11 sequence) and we repeated the experiment in this revision (see methods). Finally, BMP11 RNAi in two newly added species give results that make sense, e.g. some of its effects reflect conserved function of BMP11 across the three species.*

Yet, none of them provide formal support to the conclusion from the manuscript title "pleiotropy promotes male exaggerated weapon"; this is rather a reasonable hypothesis that can be derived from the results. Note that the story of BMP11 being an example of a "magic" gene that may influence several traits that all need to evolve simultaneously under sexual selection might very well be true, but the results presented in this paper do not prove that it is the case (and even less that BMP11 actually evolved under sexual selection in this species).

***Authors’ response:***

- *Our RNAi knockdowns show that BMP11 is required for three traits the sum of which distinguishes Microvelia longipes from the other species in the Microvelia genus (see new figure 3 and Toubiana and Khila 2019). These are namely a larger mean body size, exaggerated leg length, and extensive fighting behavior. This means that this gene contributes to all these traits and that is what we mean by pleiotropy. We changed the title to capture the general story of the paper.*
- *Regarding the evolutionary scenario, we now conducted the analysis of the role of BMP11 in two sister species of M. longipes. The new data are compelling and support the conclusion that this multi-facetted role of BMP11 evolved in the lineage leading to M. longipes.*
- *In the light of this new set of data, we maintain the discussion of the possible importance of pleiotropy in explaining the evolutionary stasis of allometric coefficient observed across lineages (Voje et al. 2014; Houle et al. 2019). To avoid any ambiguity regarding the hypothetical meaning of our argument, we removed the terms “genetic architecture” (l.54) and “genetic basis” (l.69) in the aims of the introduction and added a sentence explaining the need of further comparative studies of scaling relationships to have general conclusions on this topic (lines 247-249).*

I am a bit concerned that by trying to juxtapose results of different strengths (especially by including transcriptomics results that are difficult to interpret in the frame of the evolution of allometry) to address a more ambitious question, the authors may have weakened the paper.

***Authors’ response:*** *Agreed! We simplified the transcriptomic results and put some of the data necessary to justify the functional work in the supplement. As things stand now, what is left of the transcriptomic section will allow the reader to understanding where Ubx and BMP11 came from. The details of the comparative transcriptomics are part of the companion paper, with a focus on sex-biased gene expression, being evaluated elsewhere and that now is independent from this one.*

4. Some methodological details of the paper make me uncomfortable

4.1. The exclusion of putative "unaffected RNAi" individuals from phenotypic measurements (line 299) is dubious. Sup fig 10 clearly shows that pronotom/mesonotum ratios are not so good at discriminating, and no indication about the correlation between this ratio and body size is provided. Validating the exclusion by checking that excluded phenotypes behaves as control individuals looks rather circular, and I think the authors should consider measuring more accurately the size of the bias induced by such a sorting procedure. Not knowing the number of removed individuals is a bit stressful here -- due to the lack of details, here again I will have to assume that this was reasonable.

***Authors’ response:***

- *We performed new RNAi injections throughout the summer and fall to address the reproducibility and efficiency concerns raised by this reviewer and others. This allowed us to resolved this problem. We no longer need to be concerned by these wild type-like individuals so we included all injected individuals in the analysis.*
- *This said, and RNAi by nature being known to cause partial not complete reduction of transcript, we do know that some individuals will not be affected and others will be only mildly affected (see for example Khila et al. 2012). This means that non-affected individuals will cause the analysis to under-estimate the effect of BMP11 on slope. With all individuals slope is reduced by a third. We did a test where we removed WT-like individuals (just to check but we did not include this here) slope was reduced almost by half.*

4.2. There is an issue with the behavioural results: most of the observed differences are not significant. However, when looking at figure 4, the distributions of observations are totally non-overlapping, which makes me think that the statistics are flawed, and that the authors' approach is deeply under-powered. My interpretation is that the authors have recorded four replicates of the behavioural experiment involving five females and ten males for each treatment. During each experiment, many fights have been recorded (if the cryptic "frequency" Y axis in fig 4A means "number of fights", then almost a thousand fights were recorded). But in the t-test for fight duration, it seems that the authors have just included the average duration of fights, as if there was only one fight in each experiment, and n=4 observations per replicate. I think that a much better statistical framework would be to run a generalised linear model with a Poisson family for the number of fights (fig 4A), which should increase the power of the test (no need to estimate the variance for count data, as the variance is expected to be the same as the mean); the duration of fights should be analysed based on individual fights, possibly considering the replicate as a random effect to account for possible batch effects. This should definitely display significant differences across treatments, and avoid highlighting a dubious difference between "non significant" and p = 0.08 (cf figure 4B).

***Authors’ response:*** *We reanalyzed the data using generalized linear model for the frequency and duration of fights. We used glm with gaussian distribution for the frequency of fights and changed figure 4A by the actual values of fight frequencies. For the duration of fights, we also used a glm with gaussian distribution, considering all individual fight durations rather than the average values per condition. We also accounted for replicates as possible batch effects. We reported all p-values in the revised Figure 4 and corrected the main text accordingly. We also added Supplementary tables 8 and 9 that provide the record of the number and duration of all fights and a summary description of the video record timing, average fight duration and fighting frequency for each replicate.*

4.3. I found the interpretation of transcriptomics results unclear and questionable. The authors found a convincing "legs" effect (fig 2A) in both sexes from the full expression pattern, but the fact that they propose two independent analyses for males and females suggests that the pattern might be confusing when both sexes are considered together (in this context, I don't understand how the authors could conclude that the first axis is similar across sexes line 101, but I might have misunderstood something). Then they isolate genes that are over or under-expressed in the third legs in both males and females, which (as almost always in transcriptomics studies) generate a lot of sets of genes with little overlap.

***Authors’ response:*** *Here again, please note that this section is now reduced greatly. In the PCA analysis (now supplementary figure 2) we aimed to separate sexes to only focus on the leg effects and observe whether a difference existed when we compare the two sexes separately. A similar analysis was performed on our companion paper where the PCA analysis was done on the whole dataset, without separating sexes. Here we found that the first explanatory variable was the line effect, followed by the sex effect and ultimately the differences between legs. In the paper, because the PCAs were performed independently in males and females we cannot compare them (as also noticed by reviewer #2) and we therefore removed this comparison from the text.*

*We also removed the interpretation of the overlap of leg-biased genes between males and females. We only focused on the comparison of the number and degree of differential expression of leg-biased genes between males and females.*

Gene ontology returns different patterns in males and females, which seems rather odd, and the clustering illustrated in fig 2 does not seem to be very insightful (for instance, genes overexpressed in female third legs display the most similar pattern when comparing

male third legs and female first legs...). Some conclusions from the text (e.g. line 122, "the third legs express a considerable set of common genes between males and females") are vague (what is "considerable"?) and not supported by the figures (is it really surprising that the same genes are expressed in the legs of both sexes anyway?).

***Authors’ response:***

*The fact that we do not find exactly the same patterns in males and females is what the data show.*

*The clustering analysis was made, at first, to check whether leg-biased genes that were called “specific to one sex”, according to the Venn-diagrams, were not due to an artifact (i.e. lack of statistical power to also detect the same gene as differentially expressed in the other sex). We decided to remove this part as it adds complex information that is not relevant for the main question of the paper.*

*We agree with the reviewer about the subjectivity of the sentence "the third legs express a considerable set of common genes between males and females" and removed it from the text.*

*The goal of the Venn-diagrams was not to show that the same genes are expressed in both sexes. Among the thousands of genes differentially expressed between legs in the two sexes it is, of course, inevitable that a proportion of those overlaps. However, a hypothetical scenario to explain the male-specific exaggeration in the third legs would be that some genes are leg-biased in one of the two sexes only. Based on the Venn-diagram we selected, for the functional assays, a list of leg-biased genes sex-biased genes. The rational is that some of the genes involved in the growth of male’s third legs (slope>1) will be differentially expressed compared from one hand to male’s first legs (slope ~1) and from the other hand to female’s third legs (slope ~1).*

*** Details ***

* Line 61, "large body size and high variation": in the only other species that is analysed (e.g. black in fig 3), the body size variation seems even larger than in the focal species. The variation is legs size could simply be due to a change in allometry.

***Authors’ response:*** *This is correct. We used in the text the word “simultaneously” to make clear that only M. longipes displays all these characteristics at the same time (long legs, large body and highly variable body and leg length).*

* Line 67, and other places in the ms: the link between exaggerated weapon size and allometry is far from clear. Disproportionate organ size can either be due to a change in allometry and/or a change in size without a change in the allometric coefficient (if > 1).

***Authors’ response:*** *We do know that in M. longipes, there is a change in allometric oeficcient compared to several Microvelia spp. Tested here (Toubiana and Khila Proc. B. 2019). What is meant by exaggerated weapon is within the comparative cotext between various species from the same genus. To avoid any ambiguity, when using “disproportionate growth” in the context of exaggerated traits and hyperallometry, we added the words “within individuals of the same population”.*

* Line 105: "the third legs diverged more form the first legs in males than in females": this looks in contradiction to a previous statement line 98, "we failed to detect any correlation between such variation in morphology and variation in gene expression". I am not sure to understand why selecting a set of genes differentially expressed between legs 1 and 3 generates a pattern that was not present in the full set of genes (is it just a problem of statistical power?).

***Authors’ response:*** *The reviewer refers here to two independent analyses, namely the PCA analysis and the differential expression analysis (using DESeq2). Indeed these two analyses are not similar as PCA analysis looks at global patterns of variation, taking all expressed genes into account at the same time. On the other hand, differential expression analysis using DESeq2 looks at differences in expression between two or more conditions gene by gene. We used the latter analysis to generate our list of 30 candidate genes and identify BMP11 and Ubx as differentially expressed between the third and first legs in males.*

*Moreover, we decided to be more succinct on the PCA analysis, based on comment from the other reviewers too and removed the sentence "we failed to detect any correlation between such variation in morphology and variation in gene expression" from the text.*

* Line 114, 63% of leg-biased genes were common between the sexes (fig 2C): fig 2C indicates 92+157+232=481 genes overexpressed in the first legs vs third legs, 157 of them (32%) being common between sexes. I am not sure to understand what were the reported 63%.

***Authors’ response:*** *This was a mistake and corrected now, thank you for pointing it out.*

* Line 126, "extravagant signals [...] are predicted to occur in good condition individuals": how does this observation combine with the allometric argument? If high fitness males are also larger, then it is perfectly compatible with the fact that there is positive allometry involved (in which case the fitness argument is not really central, since body and organ sizes are constrained by the allometric relationship, and the association large weapon <-> high fitness is unavoidable). If this argument ignores allometry, then it means that organ size is (at least partly) independent from body size, and this suggests that the main question about allometry is not relevant (allometry becomes a statistical association which does not derive from biological constraints).

***Authors’ response:*** *In a sample of Microvelia species, we know that males can be large without evolving trait exaggeration. In M. pulchella, there is high variation in body size among males but with no leg exaggeration. This is the case even though M. pulchella males also fight with the legs (to a much lower extent though). So we are not as unequivocal as the reviewer is about the idea that hyperallometry is inevitable if large males enjoy higher fitness. What’s is striking in our results is that we could link several traits that are likely to increase male fitness to BMP11 (i. e. large body, disproportionate leg growth, fighting behavior). By this, we don’t mean this gene is magic, which would be an oversimplification. This likely means that in the lineage leading to M. longipes, whatever mutations that made it possible for these traits to evolve in the males involve BMP11 being part of a developmental genetic mechanism that make these phenotypes correlated.*

* Line 144: I am not sure to understand why BMP11 is defined relative to its vertebrate homolog (including in the full phylogeny of Sup fig 7), there is no ortholog in insects (including D. melanogaster?).

*Autho****rs’ response****: We did not find a true ortholog even in D. melanogaster which has three BMP-type ligands: Decapentaplegic (Dpp), a functional ortholog of vertebrate BMPs 2 and 4, Glass bottom boat (Gbb), a member of the BMP 5,6,7 subgroup, and Screw (Scw), a distantly related family member (Newfeld et al., 1999).*

* Line 168 and 192: good practices in statistics require to report full p-values (and not only p < 0.05, which makes meta-analyses more complicated).

***Authors’ response:*** *This has been corrected*

* Line 209: this is an interesting (but confusing) use of "plastic response", as no environmental response is involved here.

***Authors’ response:*** *We replaced “plastic response by “adaptive response” which states possible broader mechanisms than an environmental response*

* Line 255: there is no fig 5C.

***Authors’ response:*** *This has been corrected.*

* Line 240: I am confused by the claim that trait exaggeration evolves despite the evolutionary stasis of slope. This is clearly not the case here.

***Authors’ response:*** *The evolutionary stasis of slope is a conclusion made from a meta-analysis and it is a statement made relative to the evolution of body size and intercept which evolve faster (Voje et al. 2014; Houle 2019). The fact that allometric slope is a trait that generally evolves slower than body size and the intercept does not mean that slope cannot evolve. In the Microvelia taxon, M. longipes clearly was able to evolve allometric coefficient (slope > 3 is pretty large). Still, M. longipes is the only known Microvelia (among some 200 known Microvelia spp.) that show this pattern.*

* Line 262, "analysis of variance", do you mean "principal component analysis"?

***Authors’ response:*** *This has been corrected*

* Lines 350, 361: in the references, some author names are not capitalised.

*Autho****rs’ response****: The references are automatically formatted using EndNote.*

* Figure 2, I found the Venn diagrams rather confusing (the overlapping part is not proportional, and the diagrams scale is different in fig B and C. All diagrams seem to be scaled relative to the female count, which does not seem very intuitive). The color scale (low <-> high) is particularly uninformative.

***Authors’ response****: We changed the proportional size of the Venn diagrams (keep them all the same size) and removed the color scale that was linked to the heatmaps.*

* Fig 3: state that Mpul stands for M. pulchella in the caption of figs 3A and 3B. It would be great to have the colour code indicated in the figure (perhaps replacing the R^2, which is not informative in a figure since it is easy to eyeball the fit?).

***Authors’ response:*** *We fixed these issues throughout the figures.*

* Fig 4A: "frequency of fights" is unclear (is it "number of fights"?).

***Authors’ response****: We now specify in the legend “frequency of fights (number of fights per unit of time)”.*

**Reviewer #2:**

This manuscript was coreviewed by a PI and a postdoc.

Toubiana et al. provide here a very interesting study that combines descriptive, observational data about gene expression with experimental, functional data about the developmental roles of important genes. Their results show a very satisfying connection between the development of an exaggerated sexually dimorphic trait and on male behavior, indicating a pleiotropic role for one of the genes studied, BMP11.  Although not yet understood mechanistically, this link between behavior and morphology is expected to have interesting evolutionary consequences.  The authors have done a fine job tying their results to outstanding questions about the major hypotheses relating to the evolution of exaggerated weaponry.  However, we had to struggle in multiple places to figure out what analyses were done and thus we believe that the manuscript would benefit from clarification of some key components prior to publication.  So modified, this would make it an appropriate and very accessible article for PLoS Biology's broad audience.

Our substantive concerns with the paper are primarily about places where the paper does not make clear what comparisons are being discussed and what exactly the underlying data are.  These concerns likely stem in part from the short format of the manuscript, in which methods have been very condensed.

***Authors’ response:*** *We have now tried to simplify and clarify these ambiguities throughout the paper. Please note that we shortened the comparative transcriptomics section at the request of the editor to allow this paper to stand apart from its sister companion genome paper that is being revised elsewhere.*

Data availability:

Please ensure that the bioproject PRJNA610161 is the correct accession number and that the project is made public by the time of publication. As of 4/27/20, this accession number does not resolve to a public project. (You can release the project without releasing the data.)

Additionally, we did not have access to the supplemental tables and thus we were unable to check whether they included the data we would expect—i.e., tables that include the data necessary for reanalysis of both allometry and gene expression data.  (A request was submitted to the PLoS Biology team on 4/24/20, and we received a response that the request has been forwarded, but we have not received the tables; we did get to enjoy the supplemental videos and figures.)

***Authors’ response:***

- *Accession number: We now made sure the project is public, thank you for pointing that out. The project ID is* PRJNA642372*, and we requested it to be released immediately. The reviewer can also access the data through this link:* [https://dataview.ncbi.nlm.nih.gov/object/PRJNA642372?reviewer=fiqtv1ej9ndtflp0v5h74n37dj](https://dataview.ncbi.nlm.nih.gov/object/PRJNA642372?reviewer=fiqtv1ej9ndtflp0v5h74n37dj)
- *Supplementary tables: This incident was truly unfortunate (see how this happened above). Yes, indeed PLoS Biology notified us and asked for the tables on April 29. We sent the tables via a link the same day and we were notified that the tables were downloaded by PLoS Biology again the same day. We do not know why the reviewers still couldn’t see them. All tables are available now.*

Aims/analyses/interpretations:

Phrasing of main aim: "determining the genetic architecture) (l. 54) and "determining the genetic basis (l. 69) both lead readers to expect an approach that identifies causal genetic variation, whereas the research approach used here identifies genes that are differentially expressed and functionally important, but may or may not be the loci responsible for the evolutionary changes (even in the context of these selected lines).  Thus, we suggest rephrasing the aims.

***Authors’ response:*** *Yes agreed! We now rephrased those passages to read like this: “Determining the genes that functionally influence allometric slope” and farther in the text: “to determine the genes regulating the development of the third leg exaggeration in this species”.*

Experimental design: The methods should briefly state the sampling scheme for the transcriptomic data: i.e., that the starting material was lines selected for larger differences in leg size, and that gene expression in 2 lines * 2 sexes * 3 replicates *3 legs were characterized, rather than refer to the companion manuscript for it..

***Authors’ response:*** *The methods section is now detailed for the paper to stand alone. This was also requested by the editor. Now we added a more detailed explanation of the experimental design in the “Comparative transcriptomics: analyses of variance” paragraph of the Material and method section.*

PCA analysis (results on l.96-102): We question the interpretation of this analysis and recommend dropping it from the manuscript.  If it is maintained, then a clearer explanation (including methods: e.g., what genes (all?  leg-biased?  different subsets in the 2 sexes?), how was PCA implemented) is needed both of what was done and of how the result supports the interpretation.   We do not think it is meaningful to compare the % of variance explained by axis 1 between males and females if these are separate, independently run analyses (which is our impression).  Even if total variation (not just %) were greater in one than the other, additional work would be needed to demonstrate that leg3 was the cause and further to demonstrate that a specific set of genes is involved (e.g., loading information).

***Authors’ response:*** *We performed the PCA analysis on males and females separately and now give more details in the Material and method (which genes used, filtering process, correction of batch effects). We also reduced substantially the result section on PCA analysis and removed the comparison regarding the % of variance explained by axis 1 between males and females. Again, please note that this section was dramatically reduced. Instead, we completed an evolutionary analysis of BMP11 function by including two additional sister species.*

Differential expression analyses (results on l. 103-123; methods on l. 267-275):

It was difficult to figure out which comparisons were used to identify genes as 'leg-biased', even after having read the relevant sections of the companion paper. We concluded that the analyses were done on pairs of legs (1 vs. 3 and 1 vs. 2) from 2 lines * 3 replicates in each sex separately.  If that's correct, then including sampling design in methods and changing l. 103-105 as follows would clarify:

"To identify the genes underlying the growth differences in serial homologs, we identified genes that were differentially expressed between leg pairs (first vs. third leg and first vs. second leg) in each sex separately."

***Authors’ response:*** *We detailed the differential expression analysis and what ‘leg-biased’ means in both the Result and Material and method sections. We also added in the Material and methods the sentence proposed by the reviewers.*

(While the term 'leg-biased' is parallel to 'sex-biased', it may be more prone to being misunderstood as implying a comparison between legs and some other tissue.  Thus, we wonder if this term could be avoided—but we didn't come up with a good alternative.)

***Authors’ response:*** *We also struggled with this. We went through the manuscript and inserted explanatory text in brackets to make sure the reader follows.*

We remain unclear on what the set of "upregulated genes restricted to male third legs" is; we could not match the possibilities we considered for the number of genes given (489) and were unclear what 'restricted' means here. Figure (2B) shows 273+56=329 genes in the Venn diagram up regulated in the male T3 leg. Thus, we would have expected the 489 to be either 329 + 56 or 329 or some smaller number if genes upregulated in other contexts (e.g., male T2) didn't count.  Is this a typo, or are you referring to a different defined set of genes in the text versus the figure? Clarifying this should also help clarify what is meant by the statement on l. 109-111 that the male regulated genes are "similarly expressed" in females.

***Authors response:*** *In the text and in the hierarchical clustering analysis “upregulated genes restricted to male third legs” refer to the 273 genes that are upregulated in the third legs only in males. We have now corrected this mistake, thank you for pointing it out.*

(This hierarchical clustering result may be more or less forced by the way in which genes were chosen for analysis—i.e., the set of genes displays maximal differences between male T1 and T3 (and perhaps isn't DE between female legs?  Despite the clustering

pattern, Figure 2B (right) gives an overall impression that expression levels are not similar between female T1 and T3).

***Authors’ response:*** *The hierarchical clustering analyses was indeed forced towards the genes that were up or downregulated in either males or females only. For example, in the then Figure 2B (right) we selected genes upregulated and restricted to male third legs (273 genes). We can see how this could create confusion. We have now removed the clustering analysis and replaced it with an MA plot and maintained the Venn Diagrams (new Supplementary Figure 3B).*

l. 114: where is the 63% figure coming from.  Based on figure 2C, I would have expected the figure to be 157/(92+157+232) = 33%

***Authors’ response:*** *63% was coming from the female side only (157/(92+157). We corrected this mistake and modified the calculation in both third and first legs accordingly.*

Supp. Fig. 2 (and associated ANCOVA test on l. 83): We would expect that development time was potentially predictive of leg length, and thus that it should be on the x-axis (rather than the other way around).  Also, if the ANCOVA was applied to this analysis, please make clear what the covariate was.

***Authors’ response:*** *We reversed the two axes and applied the ANCOVA accordingly. The conclusion remains unchanged. We also added in the text, line 134, that body length was used as covariate.*

We encourage a supplemental methods section that covers in more detail the methods you're referencing from the Genomics companion paper in supplemental methods for this paper. Yes, it's duplicative, but if someone has access to one and not the other, they will thank you for the duplication.  Additionally, sharing the analysis code would be helpful.

***Authors’ response.*** *Agreed! As initially the two papers were submitted back to back, having details in the other one made sense. Now that they are in separate journals, we agree that each of them needs to stand alone. We added those details in the supplement.*

l. 265 (and elsewhere): Please include the R package name and the version number in the main methods text for all R packages used, as was done for pvclust (including also the version of R, which is separate from the version of RStudio).

***Authors’ response:*** *We included the names and versions of the R packages ‘ade4’ (used for Within Classe Analysis), ‘DESeq2’ (used for differential expression analysis), ‘smatr’ (used for statistics in allometries), ‘TopGO’ (used for GO terms analysis) and ‘goeveg’ (used for the calculation of coefficients of variation).*

Figure 2 comments: We recommend that in genes be sorted by expression values for the focal leg type. For example, if the male heatmap in 2B were sorted by expression of T3 leg (high > low) it would be much more obvious that the female T3 leg expression is very different from male T3 leg.

***Authors’ response:*** *We have decided to remove the heatmap as it created confusion.*

Minor points/typos, etc.

1)    Line 278: How many genes were included in this hierarchical clustering analysis?

***Authors’ response:***

*We have decided to remove the heatmap and the hierarchical clustering analysis as it created confusion.*

2)    Line 300-301:   Since you are discarding some data points (legitimately!) I recommend including the percentage of injected adults that had the short pronotum phenotype versus those that didn't for both controls and experimentals.

***Authors’ response:*** *We repeated the RNAi experiments and resolved the problem of the wild-type like individuals. We now include all individuals in the analysis. Including WT-like samples under-estimates our results, but it is best to be conservative. All the raw data with information about pronotum/mesonotum sizes etc are available in supplementary table 7. Of note, we now have the same effect on pronotum in one of the new species we added. In the third one (M. pulchella), this trait does not apply, as the pronotum does not cover the mesonotum. BMP11 RNAi in M. pulchella has no effect on pronotum as one would expect.*

3)    Lines 127-129 ("Proposed mechanisms for the development of exaggerated phenotypes include increased sensitivity or the production of growth factors by the exaggerated organ [4, 25].") That sounds to me like you're talking about two distinct hypotheses. But it seems like the next sentence is referring to a single hypothesis ('this hypothesis'). Please clarify—it may be as simple as changing 'this hypothesis' to 'these hypotheses'

***Authors’ response:*** *done!*

4)    Lines 150-166: To make this more accessible to a general bio audience -- In these sentences that sum up the results, can you rephrase what change in intercept and coefficient means in terms of relative and absolute sizes of legs and bodies?

***Authors’ response:*** *We added two sentences “(i.e. increases the relative size of the third and second legs in a disproportionate manner in small and large individuals)” and “(i.e. increases the relative size of the third and second legs in proportionate manner across individuals)” to explain to effects of BMP11 and Ubx.*

5)    Line 156-158: Perhaps rephrase sentence as follows  'The allometric coefficients for the first and second legs of males were alsso lower than in the corresponding controls, but the changes were smaller than for the third legs.'

***Authors’ response:*** *We rephrased the sentence as per the reviewers’ suggestion.*

6)    Line 164: Should be "....closer to those of its sister species..."

7)    Line 230: "Studies of scaling relationships have reported" rather than just "reported"

8)    Figure 1 - Line 439 "Power low regression" should be power law regression.

9)    Figure 4: - legend, the word "defects" should be "effects".

***Authors’ response:*** *All these points were corrected according to the reviewers’ recommendation.*

**Reviewer #3:**

Review of Toubiana et  al

This is a really nice and very interesting paper describing the role the BMP and Ubx play in regulating the morphological scaling of the third leg in male water strider of the species, Microvelia longipes. Males of this species use their exaggerated third legs to compete for access to females. The authors used transcriptomics to look at differences in gene expression between the isometric first and hyperallometric third leg, in males and females. Using these data they constructed a list of 30 candidate genes that they functionally tested using RNAi. They found that two, BMP11 and Ubx had an effect on scaling. Specifically BMP11 reduced body size in males and females, and reduced the slope of the 3rd leg-body scaling relationship in males, while Ubx had no effect on body size, but reduced the intercept of the 3rd leg-body in males and females. At the same time knock-down of BMP11 reduced the aggressiveness of males.

The paper is significant in that it is one of the few that identifies a developmental mechanism that controls the slope and intercept of a morphological scaling relationship. I certainly think that it  is of sufficient general interest to be published in PLoS Biology. Nevertheless, I have a number of suggestions about how the paper can be improved. The tables were not included in my review copy of the supplementary material, so some of my concerns may be addressed there.

1) A key finding of the paper is that knock-down of BMP11 changes the slope of the 3rd leg-body scaling relationship. Looking at figure 3C, it is possible that the 3rd-leg-body scaling relationship of  the BMP11-RNAi males may be an extension of the wild-type scaling relationship but in smaller males. That is, among small wild-type males, the slope of the 3rd-leg-body scaling relationship is shallower. If this were the case, then it  is possible that  BMP11.RNAi simply reduces body size, with no additional effect on scaling. To test this, the authors should look at scaling in males that are small, for example by reducing access to food during development. If the author's hypothesis were correct, then these small males should have the same body size as BMP11-RNAi males, but a steeper leg:body scaling relationship. These data would also confirm that the pronotum/mesonotum ratio does not increase with an increase in body size. If it does, then exclusion of BMP11-RNAi

individuals with high pronotum/mesonotum ratios from the analysis of the effects on BMP11 on 3rd-leg-body scaling is not reasonable: the authors are simply excluding larger individuals with steeper leg:body scaling.

***Authors’ response:***

- *We have done this reaction norm experiment in a recently published study (Toubiana and Khila Proc. B. 2019). We tested the effect of diet on slope using one of the inbred lines used here in addition to a non-inbred natural population. In these two populations, we were able to show that body size responded dramatically to diet treatment (poorly fed males were small and well-fed males were large). However, there was no effect of diet treatment on slope (please see Figure 4 and supplementary figure 5 in Toubiana and Khila Proc. B. 2019). Based on this understanding, we are confident that BMP11 truly contributes to allometric slope during development, and not only body size. We inserted a sentence in the result section of the current manuscript stating this. Furthermore, we now did the same experiment in two additional species. In one of them, M. sp, body size is as large as M. longipes but the allometric coefficient of the third legs is below one. In this species, BMP11 RNAi significantly reduces body size but does not affect slope (please see new Figure 3).*
- *For exclusion of WT-like individuals, this is no longer the case in the revision. We managed to solve this problem by repeating the RNAi experiments and by generating new BMP11 RNAi samples. The scaling relationships are now based on the entire sample in Supplementary table 7. This still under-estimates the effect of RNAi, but we are satisfied knowing that we are being conservative.*

2) In several places,  the authors confound the necessity with sufficiency. For example: L158: "Therefore, BMP11 increases both the allometric coefficient of all legs and mean body size in the males", and L197: "These results suggest that BMP11, but not Ubx, increases fighting frequency in M. longipes males." Both these statements argue implicitly that BMP11 is sufficient  to increase the allometric coefficient or fighting frequency. Since the authors have not increased BMP11 expression, but only decreased it, they can only be certain that BMP11 is necessary for the hyperallometry of the legs, and for increased fighting behavior, but not that it is sufficient.

***Authors’ response:*** *We fixed this potential misunderstanding throughout the manuscript.*

3) The authors do not provide evidence that BMP-RNAi or UBX-RNAi knocks down expression of both these genes. They should do so.

***Authors’ response:*** *We have controlled for this in two ways. (1) we now have generated BMP11 RNAi using three fragments of the gene and we obtain the same results (supplementary table 7). (2) we conducted qPCR and showed that RNAi reduced significantly BMP11 expression in both sexes (see Supplementary Figure 8.*

4) I did not  have access to the table showing  the statistics  used to test the hypotheses described in  the paper. They need to be included for a complete review. In particular, I am not sure how the authors tested for the effect of BMP-RNAi on variance (L150)

***Authors’ response:*** *Yes, this was an unfortunate incident and we apologize. The tables were mixed with the companion paper, which was initially submitted back to back with this one. The supplementary tables are now available with the revision.*

*We tested for the variance using F-tests and by comparing coefficients of variation on raw data using the R package ‘goeveg’ version 0.4.2.*

Minor points:

5) The section on transcriptomics of the different legs in males and females is difficult to follow. In particular, the authors do not appear to be using the data to test a clear hypothesis about how gene expression is regulated in the legs in males and females. What do these patterns of gene expression mean? would these patterns look like under different hypothetical mechanisms of leg exaggeration? What would you expect the pattern of PC1 and PC2 to look if morphological divergence in serial homologs between the sexes was the "result of global change in gene expression but rather from specific sets of genes" (L101)? The title is also misleading: "Leg exaggeration is associated with a specific signature of leg-biased gene expression". Given that only gene expression in the legs was measured, of course the gene expression is leg-biased. This section should be re-written or excluded.

***Authors’ response:*** *We have dramatically reduced this section, also at the request of the editor and other reviewers, to make it easier to understand and to allow this paper to stand alone from its sister companion paper that is being revised elsewhere (the two papers were initially submitted back-to-back to PLoS Biol.). This paragraph focuses primarily on associating gene expression with the exaggeration of the third legs in males and identifying possible candidates for testing in the following sections.*

6) Including color and shape keys in the figures would help the reader interpret the charts.

***Authors’ response:*** *We have now fixed this throughout the figures.*

7) For Figure 3, the authors should indicate what M.pul means. It took me a while to realize it meant M. pulchella.

***Authors’ response:*** *Sorry about this, it is fixed now.*
