# Supplementary material for: The growth factor BMP11 is required for the development and evolution of a male exaggerated weapon and its associated fighting behavior in a water strider
Source: PLoS Biol. 2021 May 11;19(5):e3001157. doi: 10.1371/journal.pbio.3001157 (PMC8112723; doi:10.1371/journal.pbio.3001157)
Supplement: Attachment — Submitted filename: Response_To_Reviewers_R2.docx [file pbio.3001157.s027.docx]

**REVIEWERS' COMMENTS:**

**Reviewer #1: [identifies himself as Arnaud Le Rouzic]**

Review of "The growth factor BMP11 is required for the development and evolution of a male exaggerated weapon and its associated fighting behavior" by Dr Toubiana and colleagues, PLOS BIOL MS D-20-00810R2. This is a deeply revised version of MS D-20-00810R1 I had the opportunity to review last year. My opinion about the manuscript was balanced, as I found the topic and the results very interesting, but I had major concerns:

1) a lot of data and methodological information were missing,

2) it was not entirely clear that allometry was analyzed and interpreted properly,

3) transcriptomics results were difficult to interpret and not important for the paper, and

4) there were some methodological issues with some analyses. The authors have substantially rewritten the paper and clarified many issues, both in the ms and in their response to the reviewers. The revised version is quite convincing, I liked it a lot, and I have no further reason to oppose publication.

*We would like to thank the reviewer for pointing out these issues for us to have the chance to correct them. The paper is now much improved as a result.*

My point #1 was completely addressed. The authors explained that two companion papers had to find different publication routes, and that the state of the previous version reflects an incomplete split.

Point #2 has been largely addressed. Yet, I am still not completely convinced by the authors' interpretation of what happens between the 5th instar and the adult stage. It seems to me that the authors' narrative relies on the idea that "something" (a physiological discontinuity) happens there (the authors chose to extract RNA for transcriptomics at that time point). I totally agree that third legs grow substantially between the 5th instar and the adult stage, more than between any previous developmental stages. However, my interpretation of the growth curves (fig 1B) and supplementary tables 1 and 2 is that the development is possibly allometric (difficult to confirm without reanalyzing the data including body size at each larval stage). This is probably a matter of interpretation, but if the development of legs follow an allometric trajectory, nothing "dramatic" occurs at the 5th instar beyond the fact that growth curves diverge more and more on the arithmetic scale (a mere consequence of plotting growth, which is essentially a geometric process, on an arithmetic scale). On the opposite, if the allometric trend breaks down for the 3rd leg (i.e. leg length does not follow a power law), then I would agree that the pattern is discontinuous and that something special happens here. This, of course, should be regarded as a minor conceptual disagreement between the authors and a reviewer, and does not require a change in the manuscript.

*We made some changes in the text to take this comment into account. The text now reads like this: “The length of the third legs continues to increase between 5th instar and adult males in a higher rate when compared to females’ legs or to the two other male legs during this last developmental stage (Figure 1B; Supplementary table 1).”*

The focus on transcriptomics has been largely reduced, which fixes my point #3. Finally, the authors ran new RNAi injections and ran the analysis without excluding control-looking individuals (point #4.1), redesigned the statistical analysis of the behavioral assay (point #4.2), and fixed interpretation issues from the transcriptome analysis (point #4.3). ***

Minor suggestions:

* Lines 244 and 245: it is probably necessary to indicate "allometric slope" and "allometric intercept".

* Line 321: PCA and analysis of variance are two different analyses.

*This was fixed, thank you.*

* Figures 2 and 3(M, N, O): the x and y ranges are much wider than the data range, making it difficult to read the figures. I am not sure it is justified to plot all data (across sexes, legs, and species) on the same x and y range, since direct comparison is not of interest here.

*All these suggestions have been introduced as recommended by the reviewer.*

**Reviewer #2:**

Overall, I found this revised manuscript substantially clearer and improved. It is much easier for readers to figure out what was done; the new functional data from two closely related species are an interesting addition; and the very strong effect of BMP11 on both behavior and the relationship between leg length and body size is unexpected and fascinating in the context of the evolution of sexually selected traits.

*Thank you.*

There are, however, still a few places where I think the ms. would benefit from additional clarification or where more caution in interpretation may be called for:

l. 68-69: unclear why large body size is relevant. This isn't highlighted in the background nor is it inherent to consideration of allometry. (But it's a point that's returned to as a reason this species was chosen in several places.) The relevance of large body size either needs to be explained or statements about it should be removed.

*We addressed this comment in the introduction. That passage now reads as follows: “Males of other species that exhibit either large body, such as Microvelia sp., or high variation in body size, such as Microvelia pulchella a sister species to M. longipes, do not display exaggerated leg length, suggesting that the evolution of hyperallometry may be linked to the evolution of increased mean and variation in body size [19].”*

l. 259-260: Ubx was expressed at high levels in T3 legs long before the hyperallometry evolved. So I don't think this can be taken as evidence of an alternative pathway to trait exaggeration that's being argued for here. Additionally, I don't recall evidence that BMP11 is NOT upregulated in the sister species. This would need to be the case to support this central claim that it's overproduction of the growth factor that is causing increased leg growth. The data seem equally compatible with alternative interpretations about what the evolutionary change was, such as that it's a change in the expression of something downstream of BMP11 or in the responsiveness of something to BMP11 signaling. In the absence of comparative expression information, I think greater caution in interpreting the results is warranted.

*We do agree with the reviewer about Ubx, and we deleted Ubx from line 280.*

*For BMP11, we show that expression is much higher in the exaggerated leg (third leg) compared to the body and to the other legs. Our conclusion stems from comparison within M. longipes. Still, we do not disagree with this statement by the reviewer. The sentences in line 281-283 make no reference to other species but rather clearly make comparisons between tissues of the same individual; it reads like this: “These molecules are produced in excess by the exaggerated tissue, indicating an alternative path to trait exaggeration where over-production of the growth factor occurs locally rather than through heightened sensitivity to a systemic factor.”*

Related to results interpretation, I was unclear what the relationship of the three studied species is. Among the three, if sp. nov. and pulchella are most closely related, with longipes sister to a clade that contains both of them, then the authors should be more cautious about interpretations of ancestral versus derived states (as there is not direct evidence about the polarity of a change when the difference distinguishes sister groups). One direction of change may be more plausible based on other considerations, but the distribution of traits on the tree would not be providing additional supporting evidence. (On the other hand, if one of the two additional species is more closely related to longipes, and the two additional species have the same trait value, this comment can be ignored, because the result would help polarize the change.)

*The phylogenetic relationship between M. longipes and the two other species was published here (Toubiana and Khila Proc. B 2019). M. longipes and M. pulchella are sister species. We now state this in the introduction for clarification. This also responds to the comment l. 73 below.*

Minor changes:

l. 55: clarify by writing 'Allometric slope'

*Done.*

l. 73: 'close relative' sounds more natural than 'closely related species'

*We say sister species to clarify the phylogenetic relationship between M. pulchella and M. longipes.*

l. 80: change 'multi-facetted' to 'multifaceted' (note: two corrections to word)

*Done.*

l. 94: unclear what's meant by 'fluctuation' of the growth birth. Just variation?

*Yes, we changed it to variation*

l. 98-99: unclear which similarity is meant: that there's a difference similar to the diff. between male and female 3rd legs in the 1st and 2nd legs? Or just that all these legs grow faster starting in the 4th instar?

*We clarified this and it now reads as follows: “The first and second legs in males and females also grew faster at the final instars, although their growth rate was not as extreme as the third legs (Figure 1B).”*

l. 104-105: comparison unclear: what's the global difference between? Male legs and female legs? Different legs within a sex? l. 114-115: need an only: predicted to occur ONLY in individuals in good condition because.....

*l. 104-105: We specified that the comparisons were made between third and second legs over first legs within males and between male and female third legs over first legs.*

*l. 114-115: we corrected the sentence as the reviewer suggested.*

l. 155: change male's to males'

*Done.*

l. 173-175: Not clear to me on what basis one would predict that higher expression would translate into a larger RNAi effect. I could present an argument for the opposite conclusion: that with low-level expression, knockdown may reduce expression to a greater degree, leading to a stronger effect.

*Agreed, we now corrected this by deleting this part of the sentence: “…, consistent with the effect of RNAi”*

l. 176: check number of digits in slopes; I'm guessing this should be reported as 3.1 vs. 2.0

*Done.*

l. 296: change 'effect' to 'effects' transcriptomics: this methods section omits saying what the reads were mapped against. (I assume the genome from the former companion manuscript.)

*This was also fixed.*

l. 339: unclear why 2nd vs. 3rd leg comparison is omitted.

*This was a mistake. Indeed, all comparisons were analyzed. We corrected it and mentioned about the 2nd vs 3rd leg comparison in the text.*

l. 365: should 'wo' be 'two'?

*Yes, fixed.*

l. 383: I was confused by 'For RNAi'; should this be 'For qPCR'?

*Yes, this was corrected.*

l. 410: Please specify the version of R being run by RStudio.

*Done.*

l. 565: delete 'neither' l. 575: change 'in in' to 'in'

*Done.*

l. 580: space needed in M. pul

*Fixed.*

l. 580: change 'states' to 'stands'

*Done.*

Figure 2A, Ubx female b (on graph): think value should be 0.4787

*Yes, thank you for spotting that typo.*

Fig. 2 and Fig. 3 graphs: When there is no significant difference between slopes, I think the results are better represented by providing a single estimate of b (or an estimate that applies to a single sex)

*We prefer to report the exact value that was calculated for each dataset. This might make the figure look busy, but it is accurate.*

Supplementary materials: Quality of some of the figures (e.g., S3 & S7); I suspect Word is to blame for this, and that either embedding some other way or using pdf may solve the problem.

*We fixed this and now provide the file in PDF format.*
